# A computational medicine framework integrating multi-omics, systems biology, and artificial neural networks for Alzheimer's disease therapeutic discovery

**DOI:** 10.1016/j.apsb.2025.07.018

**Published:** 2025-07-16

**Authors:** Yisheng Yang, Yizhu Diao, Lulu Jiang, Fanlu Li, Liye Chen, Ming Ni, Zheng Wang, Hai Fang

**Affiliations:** a Shanghai Institute of Hematology, State Key Laboratory of Medical Genomics, National Research Center for Translational Medicine at Shanghai, Ruijin Hospital, Shanghai Jiao Tong University School of Medicine, Shanghai 200025, China; b Translational Health Sciences, University of Bristol, Bristol BS1 3NY, UK; c Department of General Surgery, Pancreatic Disease Center, Ruijin Hospital, Shanghai Jiao Tong University School of Medicine, Shanghai 200025, China; d Nuffield Department of Orthopaedics, Rheumatology and Musculoskeletal Sciences, University of Oxford, Oxford OX3 7LD, UK; e Department of Orthopaedics, Shanghai Key Laboratory for Prevention and Treatment of Bone and Joint Diseases, Shanghai Institute of Traumatology and Orthopaedics, Ruijin Hospital, Shanghai Jiao Tong University School of Medicine, Shanghai 200025, China; f Jinfeng Laboratory, Chongqing 401329, China; g Medical Center of Hematology, Xinqiao Hospital of Army Medical University, State Key Laboratory of Trauma and Chemical Poisoning, Chongqing Key Laboratory of Hematology and Microenvironment, Chongqing 400037, China; h Bio-Med Informatics Research Center & Clinical Research Center, The Second Affiliated Hospital, Army Medical University, Chongqing 400037, China

**Keywords:** Alzheimer's disease, Systems genetics, Therapeutic discovery, Computational medicine, Artificial neural network

## Abstract

The translation of genetic findings from genome-wide association studies into actionable therapeutics persists as a critical challenge in Alzheimer's disease (AD) research. Here, we present PI4AD, a computational medicine framework that integrates multi-omics data, systems biology, and artificial neural networks for therapeutic discovery. This framework leverages multi-omic and network evidence to deliver three core functionalities: clinical target prioritisation; self-organising prioritisation map construction, distinguishing AD-specific targets from those linked to neuropsychiatric disorders; and pathway crosstalk-informed therapeutic discovery. PI4AD successfully recovers clinically validated targets like *APP* and *ESR1*, confirming its prioritisation efficacy. Its artificial neural network component identifies disease-specific molecular signatures, while pathway crosstalk analysis reveals critical nodal genes (*e*.*g*., *HRAS* and *MAPK1*), drug repurposing candidates, and clinically relevant network modules. By validating targets, elucidating disease-specific therapeutic potentials, and exploring crosstalk mechanisms, PI4AD bridges genetic insights with pathway-level biology, establishing a systems genetics foundation for rational therapeutic development. Importantly, its emphasis on Ras-centred pathways—implicated in synaptic dysfunction and neuroinflammation—provides a strategy to disrupt AD progression, complementing conventional amyloid/tau-focused paradigms, with the future potential to redefine treatment strategies in conjunction with mRNA therapeutics and thereby advance translational medicine in neurodegeneration.

## Graphical abstract

**Figure:**
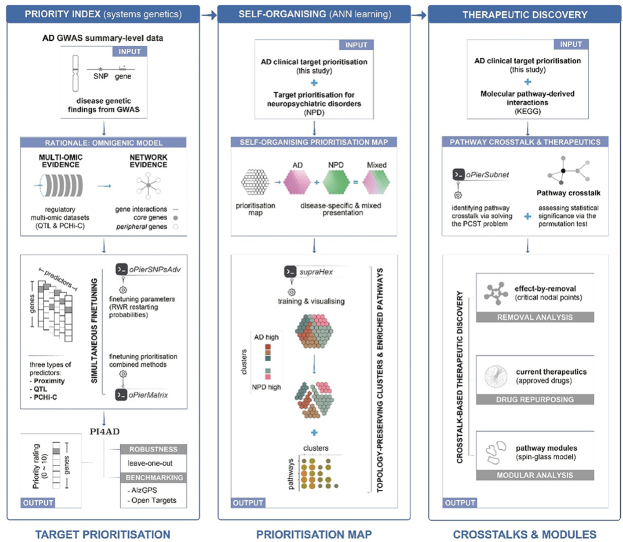

## 1 Introduction

The field of neurodegenerative disease research has continually evolved, with Alzheimer's disease (AD) representing a critical focus due to its profound impact on the ageing population. AD, a progressive neurodegenerative disorder, manifests as cognitive decline, memory impairment, and behavioural changes, severely compromising patient health and quality of life^1^^,^^2^. As the leading cause of dementia, it poses a complex challenge due to the extensive neuropathological changes^3^.

Despite advances in understanding AD pathogenesis, drug discovery and clinical trial design remain challenging. Patient heterogeneity—variation in symptoms, disease trajectories, and genetic backgrounds—complicates the development of universal therapies. This diversity results in individuals exhibiting distinct symptom profiles and progression rates, rendering one-size-fits-all treatments ineffective. Furthermore, the prolonged disease course, coupled with the challenge of ensuring drugs effectively cross the blood–brain barrier (a semipermeable membrane restricting substance entry into brain tissue)—though potentially overcome by lipid nanoparticle-mediated mRNA delivery^4^^,^^5^—complicates these hurdles further. Consequently, AD drug development is costly and time-consuming, with no disease-modifying therapies yet available^3^. Current treatments, such as aducanumab and lecanemab, provide only limited symptomatic relief, underscoring the urgent need for novel therapeutic strategies^3^.

In response to these challenges, drug repurposing and combination therapies have emerged as promising avenues. Repurposing existing drugs to modulate AD-related pathways or symptoms holds potential to accelerate therapeutic discovery^6^^,^^7^. Similarly, combination therapies—employing agents with complementary mechanisms—may enhance treatment efficacy^8^^,^^9^. For instance, anti-ischemic stroke candidate drugs demonstrate dual neuroprotective properties and improve cerebral blood flow^10^, a mechanism relevant to neuronal activity's role in glymphatic clearance (a process that removes brain waste and is implicated in reducing AD risk)^10^. TREM2-targeting antisense oligonucleotides exemplify this approach, enhancing microglial phagocytosis while suppressing chronic activation and neuroinflammation, thereby improving AD pathology and cognition^11^.

Building on these therapeutic strategies, advances in bioinformatics have catalysed avenues for drug discovery and repurposing in AD. Computational methods now integrate multi-omics data and systems biology to identify therapeutic targets. For instance, Duncan et al.^12^ utilised genome-wide association study (GWAS) and single-nucleus RNA sequencing data to identify cell-type-specific associations in schizophrenia and related psychiatric disorders (including AD), revealing potential therapeutic targets. Siavelis et al.^13^ combined multiple drug repurposing tools to prioritise anti-AD candidates, while Taubes et al.^14^ established apolipoprotein E (*APOE*) genotype-dependent transcriptomic signatures for screening *APOE*-related AD drugs. Zhou et al.^15^ further demonstrated multi-omics utility in AD biomarker discovery.

The advent of artificial intelligence (AI) has further transformed AD drug discovery. AI techniques, including artificial neural networks (ANNs), analyse complex datasets more effectively than traditional methods^16^, ^17^, ^18^, enabling applications such as network-based deep learning and graph neural networks for AD target identifications^19^, ^20^, ^21^, ^22^, ^23^, ^24^, ^25^, ^26^, ^27^, ^28^, ^29^. However, a critical gap persists: existing approaches lack systematic integration of multi-omics data with systems biology to prioritise targets and repurpose drugs. Recent progress in ANNs offers new opportunities; neuroimaging-centric models, such as prior-guided adversarial learning with hypergraph (PALH)^30^ and diffusion-based graph contrastive learning (DGCL)^31^, excel in biomarker discovery using structural and functional imaging data. However, their focus on neuroimaging contrasts with the need for multi-omics integration in therapeutic development.

**Figure 1:**
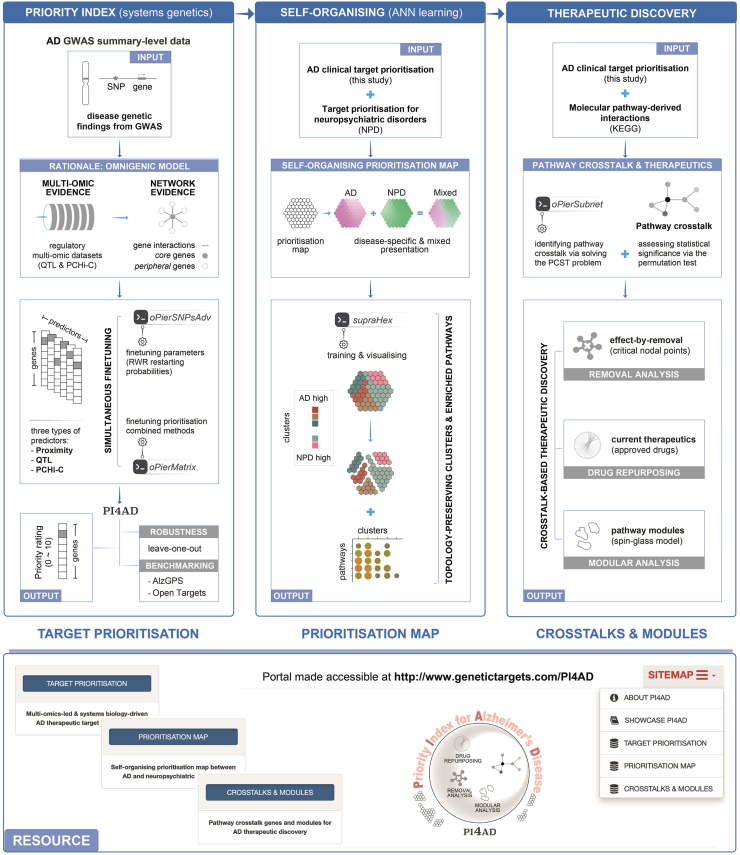
Schematic overview of the PI4AD framework for Alzheimer's disease (AD) therapeutic discovery. PI4AD integrates multi-omics data, network analysis, and prioritisation into three modules. The *clinical target prioritisation* module processes AD genome-wide association study (GWAS) summary statistics, leveraging multi-omic evidence (such as QTL and PCHi-C) and network interactions to identify core and peripheral genes, prioritised *via* a meta-analysis-like method and optimised random walk with restart (RWR) parameters. The *self-organising prioritisation map* module employs an artificial neural network (ANN) to cluster genes into topology-preserving hexagons, enabling disease-specific pathway enrichment analysis. The *pathway crosstalk* module utilises the prize-collecting Steiner tree (PCST) algorithm to identify interconnected high-priority genes, followed by therapeutic discovery steps such as removal analysis, drug repurposing, and modular analysis. The landing frontpage of the PI4AD portal hosting the resultant resource is illustrated beneath. Abbreviations (sorted alphabetically): AD, Alzheimer's disease; ANN, artificial neural network; GWAS, genome-wide association study; PCST, prize-collecting Steiner tree; PCHi-C, promoter capture Hi-C; QTL, quantitative trait loci; RWR, random walk with restart; SNP, single nucleotide polymorphism.

To bridge this gap, we present the Priority Index solution for AD (PI4AD; Fig. 1), a computational medicine tool that integrates multi-omics data, systems biology, and ANNs. Guided by a multi-omics-led and systems biology-driven principle, PI4AD addresses three objectives: (i) *clinical target prioritisation*—validating prioritisation efficacy by recovering clinical proof-of-concept targets; (ii) *disease-specific targeting*—employing ANNs to distinguish AD from neuropsychiatric disorders *via* self-organising maps; and (iii) *pathway crosstalk analysis*—identifying critical nodal genes (*e*.*g*., *HRAS* and *MAPK1*) and repurposable drugs through systems biology. By focusing on Ras-centred pathways—implicated in synaptic dysfunction and neuroinflammation—PI4AD complements amyloid/tau-centric approaches, offering an alternative strategy to disrupt AD progression. This framework establishes a systems genetics foundation for drug target discovery, guides rational repurposing, and advances translational medicine strategies for AD, with the potential to significantly impact neurodegeneration research.

## 2 Materials and methods

### 2.1 GWAS summary-level data

European population GWAS summary-level data for AD^28^^,^^29^^,^^32^, ^33^, ^34^ were obtained from the NHGRI-EBI GWAS Catalog^35^ using the Experimental Factor Ontology term ‘MONDO:0004975’. Lead SNPs (*P*-value <5 × 10^−8^) and linkage disequilibrium (LD) SNPs (*R*^2^ ≥ 0.8) were extracted. SNP scoring (Eq. (1)) integrated GWAS *P*-values, significance thresholds, and LD strength (*R*^2^):

(1) $$S_{\text{SNP}}=R^{2}\times \left(\log_{10}\frac{1-{\;P}_{\text{SNP}}}{P_{\text{SNP}}}-\log_{10}\frac{1-5\times {\;10}^{-8}}{5\times {\;10}^{-8}}\right)$$

Where $S_{\text{SNP}}$ and $P_{\text{SNP}}$ denote the SNP score and *P*-value, respectively.

### 2.2 Core and peripheral genes identified to prepare the gene-predictor matrix

Core genes were identified using genomic proximity (Eq. (2)) and quantitative trait locus (QTL) and promoter capture Hi-C (PCHi-C) datasets^36^, ^37^, ^38^, ^39^ (Eqs. (3) and (4)).

(2) $$S_{\text{nearby}\;\text{gene}}=\max_{\text{SNP}\in \text{Ω}}S_{\text{SNP}}$$

Where $S_{\text{nearby}\;\text{gene}}$ represents the score for nearby genes, $S_{\text{SNP}}$ signifies the SNP score, *Ω* denotes collections of lead/LD SNPs located within 20 kb of the nearby gene, and max denotes the maximum scoring scheme (keeping the most informative one when multiple SNPs are located within the same genomic region).

(3) $$x=-\log_{10}\left(S_{\text{SNP}↔\text{Gene}}\right)$$

(4) $$S_{\text{QTL}\;\text{gene}}\;\text{or}\;S_{\text{conformation}\;\text{gene}}=\max_{\text{SNP}\in \Omega }\left[S_{\text{SNP}}\times \text{eCDF}\left(x\right)\right]$$

Where $S_{\text{QTL}\;\text{gene}}$ signifies the score for QTL genes, $S_{\text{conformation}\;\text{gene}}$ is the score for conformation genes, $S_{\text{SNP}}$ is the SNP score, $S_{\text{SNP}↔\text{Gene}}$ denotes the strength or significance level linking an SNP to a gene (that is, the significance level of genetic association with gene expression for an eQTL dataset or protein abundance for a pQTL dataset, and the physical interaction strength with a gene promoter for a PCHi-C dataset), *Ω* stands for collections of lead/LD SNPs, and *max* denotes the maximum scoring scheme.

Peripheral genes were identified *via* the random walk with restart (RWR) algorithm on protein interaction networks (the STRING database^40^). The RWR steady-state probability (Eq. (5)) quantified gene affinity scores (ranging from 0 to 1) to seed core genes:

(5) $${\vec{P}}_{t+1}=\left(1-\gamma \right)\times A\times {\vec{P}}_{t}+\gamma \times {\vec{P}}_{0}$$

Where γ signifies the restarting probability governing the jump back to a seed node (accordingly, 1−γ is the probability of moving to a neighbour), *A* denotes the normalised Laplacian adjacency matrix associated with the network, ${\vec{P}}_{0}$ is the starting probability vector that contains seed gene scores and 0 for non-seed genes, and ${\vec{P}}_{t}$ stands for the probability vector that the walker visits network nodes at iteration *t*. This probability vector signifies the steady state and encapsulates the affinity score of all nodes in the network to the starting seed nodes once stability is achieved.

In the context of each dataset (namely, genomic proximity, QTL, and PCHi-C), the aforementioned process generated a predictor. This predictor encompassed both core and peripheral genes, along with corresponding affinity scores. These affinity scores served as a quantitative measure of the genes’ network connectivity to the seed core genes. It was observed that genes demonstrating greater network connectivity to the seed core genes were assigned higher affinity scores. Typically, seed genes exhibited a higher propensity to receive elevated affinity scores in comparison to non-seed peripheral genes. However, it is important to note that a peripheral gene could also attain a high affinity score if it manifested substantial connectivity to a significant majority, if not all, of the seed genes. Consequently, non-seed peripheral genes with relatively high affinity scores were indicative of genes that were substantially influenced by the network. To summarise, the multi-step scoring protocol, which involved the progression from lead/LD SNPs to core genes and then to peripheral genes, culminated in the formation of a gene-predictor matrix. The rows of this matrix corresponded to genes, incorporating both core and peripheral varieties, while the columns represented predictors that embodied diverse forms of evidence. The matrix incorporated affinity scores, which comprehensively integrated information from both multi-omic and network evidence sources.

### 2.3 Clinical proof-of-concept targets

To define clinical proof-of-concept targets, we retrieved information on current therapeutics from the ChEMBL database^41^. We identified ‘clinical proof-of-concept targets’ as therapeutic genes targeted by drugs in development phase III or later for AD. These targets signify substantial evidence of their efficacy in treating the respective disease and were used to evaluate the performance of our target prioritisation methods.

### 2.4 Target prioritisation and performance evaluation

We evaluated the performance of our target prioritisation methods by calculating the area under the curve for recovering AD clinical proof-of-concept targets. Leveraging the gene-predictor matrix prepared above, we evaluated various prioritisation schemes for combining predictors, including Fisher's, logistic, order statistic combined methods. For each column-wise predictor in the gene-predictor matrix, we first converted affinity scores into *P*-like values through eCDF based on affinity scores for all genes in this predictor (Eq. (6)).

(6) $$P_{i}^{j}=\text{eCDF}\left({\text{AF}}_{i}^{j}\right)$$

Where ${\text{AF}}_{i}^{j}$ denotes the affinity score for the *i*th gene in terms of the *j*th predictor, $P_{i}^{j}$ is the corresponding converted *P*-value, and eCDF is estimated based on all genes.

Then, for each row-wise gene, we combined these converted *P*-values across predictors using a combined method (see Eqs. (8), (9), (10) for the Fisher's combined method, Eqs. (11), (12), (13) for the logistic combined method, and Eqs. (14), (15), (16) for the order statistic combined method). Finally, we rescaled the combined *P*-value into a priority rating on a scale of 0–10 (Eq. (7)).

(7) $${\mathrm{PR}}_{i}=10\times \frac{-\log\;{\text{CP}}_{i}-{\mathrm{MIN}}_{k}^{K}\left(-\log\;{\text{CP}}_{k}\right)}{{\mathrm{MAX}}_{k}^{K}\left(-\log\;{\text{CP}}_{k}\right)-{\mathrm{MIN}}_{k}^{K}\left(-\log\;{\text{CP}}_{k}\right)}$$

Where CP_*i*_ for the combined *P*-value for the *i*th gene (*i*.*e*., CDF valued at *x*_*i*_), and PR_*i*_ for the priority rating for the *i*th gene (out of *K* genes).

(i) Target prioritisation based on the Fisher's combined method (Eqs. (8), (9), (10)).

(8) $$x_{i}=-2\sum_{j}^{J}\;\log\;P_{i}^{j}$$

(9) $$x_{i}\;∼\;\chi^{2}\left(2J\right)$$

(10) $${\text{CP}}_{i}=\text{CDF}\left(x_{i}\right)$$

Where $P_{i}^{j}$ denotes the converted *P*-value for the *i*th gene in terms of the *j*th predictor, *J* is the number of the informative predictors, $\chi^{2}\left(2J\right)$ denotes the Chi-squared distribution with *2J* degrees of freedom, CP_*i*_ represents the combined *P*-value for the *i*th gene (*i*.*e*., eCDF of the Chi-squared distribution valued at *x*_*i*_).

(ii) Target prioritisation based on the *logistic* combined method (Eqs. (11)–(13)).

(11) xi=−2∑jJ[Pij(1−Pij)]

(12) $$x_{i}\;∼\;\text{St}\left(5J+4\right)$$

(13) $${\text{CP}}_{i}=\text{CDF}\left(x_{i}\right)$$

Where $P_{i}^{j}$ denotes the converted *P*-value for the *i*th gene within the *j*th predictor, *J* is the number of predictors, St(5J+4) denotes Student's *t*-distribution with *5J + 4* degrees of freedom, CP_*i*_ symbolises the combined *P*-value for the *i*th gene (that is, CDF valued at *x*_*i*_).

(iii) Target prioritisation based on the *order statistic* combined method (Eq. (14)–(16)).

(14) $$x_{i}=P_{i}^{order}$$

(15) $$x_{i}∼\;\text{Beta}\left(J,1\right)$$

(16) $${\text{CP}}_{i}=\text{CDF}\left(x_{i}\right)$$

Where $P_{i}^{order}$ denotes the ordered version of $P_{i}^{j}$ in an ascending manner, *J* is the number of the informative predictors, Beta(J,1) denotes the Beta distribution with two parameters *J* and *1*, CP_*i*_ for the combined *P*-value for the *i*th gene (*i*.*e*., CDF valued at *x*_*i*_).

### 2.5 Robustness and benchmarking for performance comparisons

**Figure 2:**
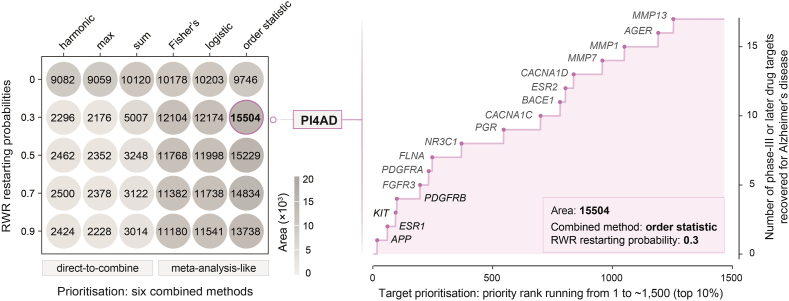
Simultaneous fine-tuning of random walk with restart (RWR) parameter and prioritisation methods. The figure evaluates the performance of RWR parameter and prioritisation method combinations in recovering *clinical proof-of-concept targets*—defined as genes linked to phase-III or later AD drug candidates. Performance is quantified by calculating the area (coloured in pink), reflecting the ability to retrieve these targets. Left panel: Heatmap comparing combined methods (columns) and RWR restarting probabilities (rows). Optimal performance (highlighted) corresponds to the *order statistic*-based meta-analysis method with an optimised RWR restarting probability (*γ* = 0.3, maximising target recovery). Right panel: Recovered proof-of-concept targets (*e*.*g*., *APP* and *ESR1*) labelled by gene symbols at optimal parameter settings.

PI4AD's robustness was assessed *via* a leave-one-out (LOO) strategy, iteratively excluding each of the three predictor types. Specifically, each predictor type was left out, and performance based on all predictors was compared to that excluding one predictor type. Benchmarking was conducted to evaluate PI4AD against state-of-the-art tools: AlzGPS (Genome-wide Positioning Systems platform for Alzheimer's Drug Discovery; https://alzgps.lerner.ccf.org)^16^ and Open Targets (https://platform.opentargets.org/disease/MONDO_0004975)^42^. AlzGPS curates clinical databases for target identification and therapeutic development, while Open Targets leverages human genetics and genomics for target validation. Performance was measured by the ability to retrieve AD clinical proof-of-concept targets, quantified using the area (illustrated in Fig. 2, right panel). For AlzGPS, prioritisations were based on: (i) clinical information (ClinVar data source ‘ClinVar_Alzheimer’); and (ii) pathogenesis evidence, including literature-derived GWAS risk genes (‘AD−inferred−GWAS−risk−genes’) and amyloid/tau endophenotypes (‘Amyloid−seed’ and ‘Tau−seed’). Notably, AlzGPS curates AD multi-omics datasets underlying AD pathogenesis (*e*.*g*., tau/amyloid endophenotype). For Open Targets, prioritisations were evaluated based on individual evidence: GWAS associations (target-disease relationships supported by significant GWAS); Gene burden (gene–phenotype relationships from rare variant collapsing analyses); ClinVar germline variants; Genomics England PanelApp (crowdsourced phenotype associations); UniProt literature/variants (protein-level functional data); Orphanet (rare disease–gene associations); Expression Atlas (disease *vs* control differential expression); and International Mouse Phenotypes Consortium (IMPC; phenotypic similarity scores). To ensure fair comparisons, network evidence (RWR algorithm with restarting probability of *γ* = 0.3) was incorporated into both AlzGPS and Open Targets. Seed genes (*e*.*g*., ‘AD-inferred-GWAS-risk-genes’, ‘Amyloid-seed’, or ‘Tau-seed’) were processed *via* RWR to generate prioritised gene lists ranked by affinity scores.

### 2.6 Self-organising prioritisation map

A self-organising algorithm in the supraHex package^43^^,^^44^ was used to construct a self-organising prioritisation map. A supra-hexagonal map was trained using the input prioritisation matrix for the top 1% genes prioritised in AD (this study) or in neuropsychiatric disorders^45^. In this map, each node *i* was represented by two types of vectors: the location vector ${\vec{r}}_{i}$ on the 2-dimensional map grid (supra-hexagonal shape), and the codebook vector ${\vec{m}}_{i}$ in the *M*-dimensional map hyperspace (here *M* = 2 as well). The training process involved two steps: (1) choosing the winner map node *w* for which its codebook vector ${\vec{m}}_{w}$ was closest to the input training vector $\vec{x}$ for genes from the input prioritisation matrix (Eq. (17)); and (2) updating the winner node *w* and its neighboring nodes by moving towards the input training vector (Eq. (18)–(19)).

(17) $$\left\|\vec{x}-{\vec{m}}_{w}\right\|=\min_{i=1,\cdots ,N}\left\{\left\|\vec{x}-{\vec{m}}_{i}\right\|\right\}$$

(18) $${\vec{m}}_{i}\left(t+1\right)={\vec{m}}_{i}\left(t\right)+\alpha \left(t\right){\times h}_{wi}\left(t\right)\times \left[\vec{x}\left(t\right)-{\vec{m}}_{i}\left(t\right)\right]$$

(19) $$h_{wi}\left(t\right)=e^{-\frac{{\left\|{\vec{r}}_{w}-{\vec{r}}_{i}\right\|}^{2}}{2{\sigma \left(t\right)}^{2}}}$$

In these equations, $\vec{x}$ represents the input training vector, ${\vec{m}}_{i}$ denotes the codebook vector for the node *i*, ${\vec{m}}_{w}$ represents the codebook vector for the winner node *w*, α(t) denotes the learning rate at training time *t*, $h_{wi}\left(t\right)$ is a Gaussian kernel function centered on the winner node *w*, σ(t) is the width of the kernel at training time *t*, and ${\vec{r}}_{w}$ and ${\vec{r}}_{i}$ correspond to the location vectors for map nodes *w* and *i*, respectively.

The codebook matrix associated with the trained map was utilised to provide a disease-specific view of target gene prioritisation. The trained map was also divided into target gene clusters in a topology-preserving manner. Enrichment analysis for genes within a cluster was based on one-sided Fisher's exact test to identify KEGG pathway enrichments. The enrichment results were reported in terms of Z-scores, odds ratio and its 95% confidence interval, and *P*-values or false discovery rate (FDR) measuring the enrichment significance.

### 2.7 Pathway crosstalk identification

Our identification of pathway crosstalk focused on genes with high ratings and strong interconnectedness. The process of identifying KEGG pathway crosstalk utilised a heuristic solution for solving the prize-collecting Steiner tree problem (detailed in Supporting Information Fig. S2)^46^^,^^47^. To evaluate the statistical significance (*P*-value) of the identified crosstalk, a degree-preserving node permutation test was carried out, with the test being repeated for 100 iterations. Moreover, the analysis provided the facility to specify a desired quantity of nodes or genes within the resultant crosstalk. This specified output was then acquired through a well-established iterative search procedure. The identified crosstalk was not only represented as a gene network but was also visualised in the form of a pathway-centric network. In this visualisation, pathways were represented as nodes, and their inferred connections were depicted as network edges. Only those pathways that demonstrated significant over-representation among the crosstalk genes, as ascertained by means of a one-sided Fisher's exact test, were incorporated as nodes. The edges were initially deduced based on the shared member genes between pathways. Subsequently, these edges were filtered by identifying MST. This filtering process ensured that only the edges present within the resulting tree were retained. Furthermore, the thickness of the edges was adjusted in a manner that was proportional to the number of shared member genes between the two endpoint pathways.

### 2.8 Crosstalk-based effect-by-removal analysis

The objective of the removal analysis was to assess the impact of nodes on the crosstalk using established concepts^48^, ^49^, ^50^. This analysis was structured in two aspects. (i) Individual node removal: This pertained to the extraction of single nodes from the network. When a particular node, which was designated for removal, held a crucial position within the network, its elimination led to a significant detachment of a considerable portion of nodes from the principal network component. (ii) Combinatorial node removal: This involved the concurrent extraction of multiple nodes in specific combinations. The aim of this approach was to identify the most favourable combinations for targeting. The intention was to maximise the resultant effect that ensued from the simultaneous elimination of particular node combinations, such as the removal of two nodes concurrently. To put it simply, the goal was to determine the combination that would lead to the largest proportion of disconnected nodes upon removal. The visual representation of the effect of node removal was achieved through the utilisation of an upset plot, with the ggupset package being employed for this purpose. This visualisation technique enabled a lucid illustration of the consequences of both single-node and combinatorial node removal, thereby providing valuable insights into the network dynamics and the significance of individual and combined nodes within the context of AD crosstalk.

### 2.9 Crosstalk-based drug repurposing

The drug repurposing analysis was carried out using data extracted from the ChEMBL database^41^, which compiles comprehensive therapeutic information on currently approved therapeutics, incorporating aspects like drugs, their developmental phases, target genes, and corresponding disease indications. In the context of our AD study, for a given disease indication, we singled out drugs that had attained approval for a specific target gene. The rationale for this selection was predicated on the target genes possessing well-articulated mechanisms of action, which could elucidate the drugs' effectiveness in treating the disease. Subsequently, these chosen target genes were clustered to constitute the drug targets category. These drug targets were characterised as genes that were the focus of any approved drugs. To test the statistical significance of crosstalk genes that were overrepresented among the approved drug targets, a one-sided Fisher's exact test was implemented. This statistical test furnished the means to ascertain the probability of observing the enrichment of crosstalk genes within the approved drug targets. Consequently, it furnished a quantitative appraisal of the significance of the association, thereby facilitating a more informed understanding of the potential for drug repurposing in the context of AD crosstalk.

### 2.10 Crosstalk-based network modular analysis

In our endeavour to identify pathway modules present within the crosstalk, we resorted to the application of a spin-glass model in combination with simulated annealing. This synergistic use of these techniques enabled us to divide the crosstalk network into discrete modules. Subsequently, for every module that had been identified, an enrichment analysis was performed. The aim of this analysis was twofold: to disclose both the functional relevance and the therapeutic potential associated with each module. The functional relevance was achieved through the identification of enriched KEGG pathways. In parallel, with a view to evaluating the therapeutic potential, we made reference to the ChEMBL database. This allowed us to identify both the enriched approved drug targets and the non-approved phased drug targets. We then proceeded to classify therapeutic targets into two separate groups. The first group was composed of approved drug targets, which were defined as genes that were the subject of targeting by any approved drugs. The second group consisted of non-approved phased drug targets, which encompassed genes that were targeted by drugs in phases of development that had not yet received approval. These two distinct target groups were subsequently employed as the foundation for conducting the enrichment analysis. By contrasting the enrichment patterns exhibited by these two groups, we were able to shed light on the clinical relevance of the network modules. For example, a higher enrichment of approved drug targets within a module might suggest a more immediate translational potential. On the other hand, the presence of non-approved phased drug targets within a module could signify areas that hold promise for future drug development and exploration.

### 2.11 Code availability

An open-source R package, available at https://github.com/hfang-bristol/PI4AD, is specifically designed for multi-omics-led and network-driven therapeutic target prioritisation in the context of AD. Together with the showcase (http://www.genetictargets.com/PI4AD/showcase), this package promotes transparency and reproducibility, allowing users to build upon our work and potentially contribute to the advancement of AD research. The PI4AD portal is accessible at http://www.genetictargets.com/PI4AD.

## 3 Results

### 3.1 Prioritisation rationale, simultaneous optimisation, and benchmarking on clinical proof-of-concept target recovery in AD

The PI4AD prioritisation framework is grounded in the omnigenic model for complex traits^51^^,^^52^, which posits that disease mechanisms involve both *core* genes (directly influencing pathology *via* multi-omic evidence) and *peripheral* genes (modulating core genes through network evidence). Building on this model, PI4AD represents a multi-omics-led and network-driven solution for AD therapeutic target prioritisation. It integrates regulatory multi-omic datasets—including genomic proximity, QTL, and PCHi-C—with gene interaction networks. The prioritisation process comprises two key steps (Fig. 1, left panel). First, predictors are generated using multi-modal regulatory genomic datasets (such as QTL and PCHi-C) and network evidence derived from the RWR algorithm. Second, these predictors are combined using meta-analysis-like prioritisation methods to rank genes by their therapeutic potential.

Central to this process is the simultaneous optimisation of RWR parameters and prioritisation methods. Through systematic evaluation of three meta-analysis-like combined methods—*Fisher's*, *logistic*, and *order statistic*—we identified the *order statistic* method with an RWR restarting probability of *γ* = 0.3 as optimal (Fig. 2 and Supporting Information Fig. S1). This configuration maximised the recovery of *clinical proof-of-concept targets*, defined as genes associated with drugs that reached phase-III or later clinical trials for AD. Applying this optimised framework to AD GWAS data, we recovered high-priority proof-of-concept targets within the top 1% of ranked genes (a total of ∼14,500 genes ranked by their priority ratings), including *APP*, *ESR1*, *KIT*, and *PDGFRB* (Fig. 2, right panel).

The target gene *APP* (ranked 18th) encodes amyloid-beta precursor protein, whose cleavage product (amyloid-beta) is a central biomarker for pre-AD monitoring^53^. Mutations in *APP* are linked to disease protection^54^, and it is targeted by amyloid-beta therapies such as aducanumab (controversial due to mixed efficacy)^38^^,^^39^, lecanemab (showing promise)^40^, gantenerumab/solanezumab/bapineuzumab (ineffective in trials)^55^, ^56^, ^57^, ^58^, crenezumab (well-tolerated but ineffective)^59^, valiltramiprosate (displaying potential benefits)^60^^,^^61^, and thonningianin A (effective in the cell model)^62^. *ESR1* (ranked 61st and renowned for its role in breast cancer^63^), encoding estrogen receptor alpha, is associated with AD cognitive and pathologic traits in women^64^ and has been investigated for neuroprotective effects *via* estrogen receptor agonists, albeit with inconclusive trial results^65^. *KIT* (ranked 95th and a potential AD target^66^) is a kinase targeted by masitinib, currently in phase-III trials for AD^67^. *PDGFRB* (ranked 100th), involved in radial glia proliferation and AD-related expression changes^54^, further underscores the framework's alignment with established AD pathophysiology. The high prioritisation of these clinically validated targets confirms PI4AD's efficacy and motivates exploration of top-ranked genes for actionable AD-specific candidates, particularly within understudied pathways (addressed in subsequent sections).

**Figure 3:**
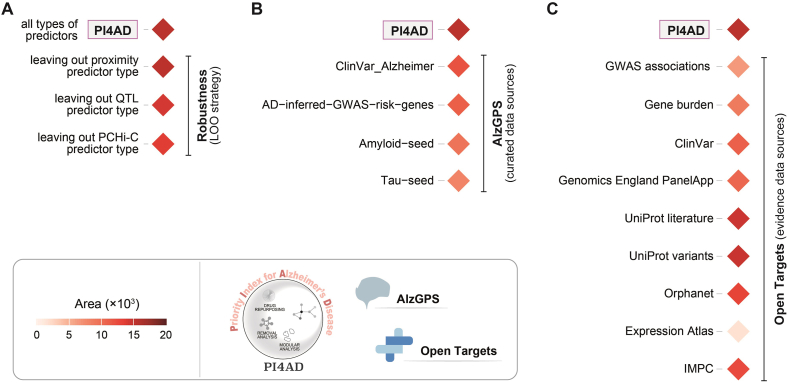
Robustness evaluation and benchmarking against competitive tools. Performance—quantified as the ability to retrieve Alzheimer's disease (AD) *clinical proof-of-concept targets*—is measured using the area, as described in Fig. 2. (A) Robustness assessed *via* the leave-one-out (LOO) strategy, iteratively excluding each of three predictor types. (B) Benchmarking against AlzGPS (Genome-wide Positioning Systems platform for Alzheimer's Drug Discovery), which curates AD clinical data (ClinVar source: ‘ClinVar_Alzheimer’) and pathogenesis data (literature-derived GWAS risk genes: ‘AD-inferred-GWAS-risk-genes’; amyloid/tau endophenotypes: ‘Amyloid-seed’ and ‘Tau-seed’, respectively). (C) Benchmarking against Open Targets using diverse evidence data sources: GWAS associations, gene burden, ClinVar, Genomics England PanelApp, UniProt literature/variants, Orphanet, Expression Atlas, and International Mouse Phenotypes Consortium (IMPC).

To validate PI4AD's robustness and comparative advantage, we conducted two analyses. First, the LOO strategy demonstrated consistent performance even after iteratively excluding individual predictor types (Fig. 3A). Second, benchmarking against established tools—AlzGPS^16^ and Open Targets^42^—highlighted PI4AD's competitive predictive validity (Fig. 3B and C). For AlzGPS, prioritisations using ClinVar and literature-derived endophenotypes (*e*.*g*., amyloid/tau pathology) were used for evaluation (Fig. 3B). Regarding Open Targets, prioritisations based on individual evidence were used for evaluation, ranging from genetic association (such as GWAS associations and gene burden) to expression and to phenotypic evidence (Fig. 3C). These analyses collectively validate PI4AD as a robust tool for AD therapeutic target prioritisation.

### 3.2 Construction of self-organising prioritisation map for AD-specific targeting in relation to neuropsychiatric disorders

**Figure 4:**
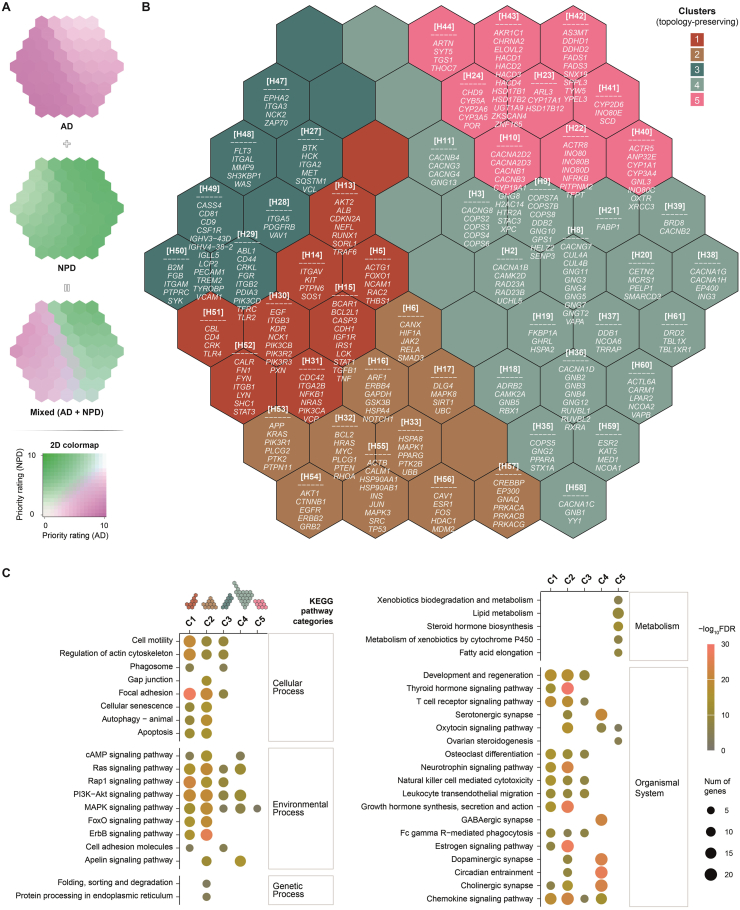
Self-organising prioritisation map constructed between Alzheimer's disease (AD) and neuropsychiatric disorders (NPD). The map was trained using self-organising unsupervised learning for the top 1% prioritised genes in either AD or NPD. (A) Colour-coded prioritisation map with two blended colours displaying disease-specific prioritisations. (B) Architecture of the prioritisation map (61 hexagons, H1–H61) divided into five clusters (C1–C5), with target genes listed in each hexagon (if any). Clustering and organisation of genes within the map visualise their relationships and groupings, highlighting disease-specific or shared therapeutic targets in AD and NPD. (C) Dot plot showing enriched KEGG pathways for each cluster. This reveals functional mechanisms and potential therapeutic targets associated with cluster genes.

To investigate disease-specific therapeutic potential, we constructed a self-organising prioritisation map using a supra-hexagonal map (Fig. 1, middle panel). This map was trained on the top 1% prioritised genes from AD and neuropsychiatric disorders (NPD)^48^, employing an ANN *via* the R package supraHex^43^. The ANN utilised self-organising unsupervised learning to cluster genes into five distinct, topology-preserving clusters (C1–C5; Fig. 4A).

Clustering analysis revealed marked differences in disease specificity: genes in clusters C1–C3 exhibited significantly higher priority ratings in AD compared to NPD, with C3 showing the strongest AD association (Fig. 4B). Conversely, genes in clusters C4–C5 were preferentially prioritised in NPD. Enrichment analysis using KEGG pathways^68^ further distinguished these clusters (Fig. 4C). Genes in C1–C3 were enriched for cell motility, focal junction, and neurotrophin signalling pathways—processes critical to synaptic plasticity and neurodegeneration. In contrast, genes in C4–C5 were functionally linked to metabolic pathways and GABAergic synapse regulation, mechanisms more aligned with NPD pathophysiology.

The above-mentioned differential prioritisation underscores AD's unique genetic architecture, highlighting cell motility and neurotrophin signalling as promising therapeutic targets. By contrast, the NPD-associated clusters implicate metabolic and synaptic mechanisms, reflecting distinct pathological drivers. These insights validate the ANN-driven map's ability to disentangle disease-specific pathways and guide targeted therapeutic strategies tailored to AD's molecular landscape.

### 3.3 Crosstalk-based therapeutic discovery in AD provides insights into Ras signalling-centred targeting potential

**Figure 5:**
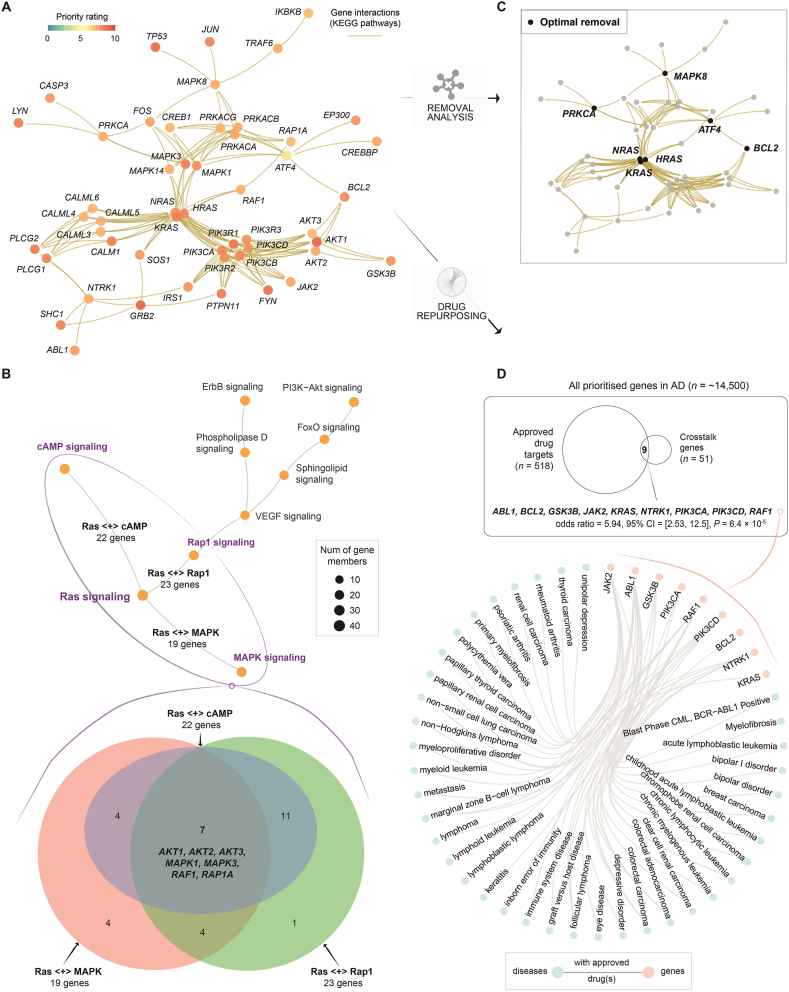
Pathway crosstalk identified for Alzheimer's disease (AD). The crosstalk was identified from pathway interactions constrained by AD clinical target prioritisation. The resulting crosstalk comprises highly prioritised, interconnected genes. (A) Gene-centric view of the crosstalk. Nodes (genes) are labelled with gene symbols and colour-coded by PI4AD priority ratings (0–10 scale). Edges represent KEGG-derived pathway interactions. High-priority Ras pathway hubs (*e*.*g*., *HRAS* and *MAPK1*) are bolded to emphasise therapeutic relevance. This visualisation enables the identification and analysis of the individual genes within the crosstalk network. (B) Venn diagram and pathway-centric view illustrating overlaps between Ras signalling and cAMP/MAPK/Rap1 pathways: ‘Ras <+> cAMP’, ‘Ras <+> MAPK’, and ‘Ras <+> Rap1’. For example, the notation ‘Ras <+> cAMP’ represents the shared genes between Ras signalling and cAMP signalling, which is further explained in the top panel. Shown on the top is a pathway-centric representation of the crosstalk, with node size indicating the number of pathway member genes. Abbreviations*:* cAMP, cyclic adenosine monophosphate; MAPK, mitogen-activated protein kinase; Rap1, Ras-related protein 1; Ras, rat sarcoma. (C) Crosstalk with the same layout as (A) but labelled only for critical nodal points/genes identified *via* effect-by-removal analysis. This focuses on the genes that have the most significant impact on the crosstalk network, and their identification can provide insights into potential therapeutic strategies that target these key genes to disrupt or modulate the crosstalk and potentially affect AD progression. (D) Enrichment of approved drug targets (from ChEMBL) within crosstalk genes. One-sided Fisher's exact test quantifies significance (*P*-value, odds ratio, and 95% confidence interval). Hierarchical edge bundling connects crosstalk genes (in pink) to approved drug indications (in cyan) *via* grey edges, thus providing a comprehensive view of crosstalk-informed potential drug repurposing opportunities.

A key feature of PI4AD is its ability to identify genes mediating molecular pathway crosstalk (Fig. 1, bottom panel), achieved through a heuristic solution to the prize-collecting Steiner tree problem (Supporting Information Fig. S2)^46^. Applying this to AD, we uncovered a 51-gene crosstalk network containing high-priority genes (*P*-value = 2.5 × 10^−4^; Fig. 5A). Pathway-centric representation of this crosstalk revealed Ras signalling as the most prominent pathway, encompassing 35 member genes (69% of the crosstalk network; Fig. 4B and Supporting Information Fig. S3). Ras signalling exhibited significant overlap with other key signallings, including cAMP, Rap1, and MAPK pathways. Among these Ras signalling genes, 22 overlapped with cAMP signaling, 23 with Rap1 signalling, and 19 with MAPK signalling. Notably, seven genes (*AKT1*, *AKT2*, *AKT3*, *MAPK1*, *MAPK3*, *RAF1*, and *RAP1A*) were common to all four pathways, highlighting their central regulatory roles (Fig. 5B, Venn diagram).

Next, we explored targetable opportunities by identifying central genes with the greatest impact on the crosstalk. More precisely, crosstalk-based effect-by-removal analysis was designed to quantify network disruption upon node/gene removal, either individually or in combination (Fig. 1, right panel). This identified seven critical nodal genes, five of which (*i*.*e*., *HRAS*, *KRAS*, *MAPK8*, *NRAS*, and *PRKCA*) are Ras signalling components (Fig. 5C). Removal of *MAPK8* alone disrupted 9.8% of network connections (Supporting Information Fig. S4). Combinatorial removal of *MAPK8* + *PRKCA* or *MAPK8* + *ATF4* increased disconnection fraction to 15.7%, while removing *MAPK8* + *PRKCA* + *ATF4* maximised disconnection to 21.6%. Strikingly, removing four nodes—three Ras signalling genes (*HRAS*, *KRAS*, and *NRAS*) and *BCL2*—disrupted 49.0% of connections, underscoring Ras signalling's centrality in AD pathogenesis.

Next, we investigated drug repurposing opportunities by mapping approved drugs for non-AD indications to crosstalk genes (Fig. 1, right panel). Nine crosstalk genes were already targeted by approved drugs (odds ratio = 5.94; 95% confidence interval = [2.53, 12.5]; *P*-value = 6.4 × 10^−5^ based on a one-sided Fisher's exact test), six of which (*i*.*e*., *ABL1*, *KRAS*, *NTRK1*, *PIK3CA*, *PIK3CD*, and *RAF1*) are integral to Ras signalling (Fig. 5D).

**Figure 6:**
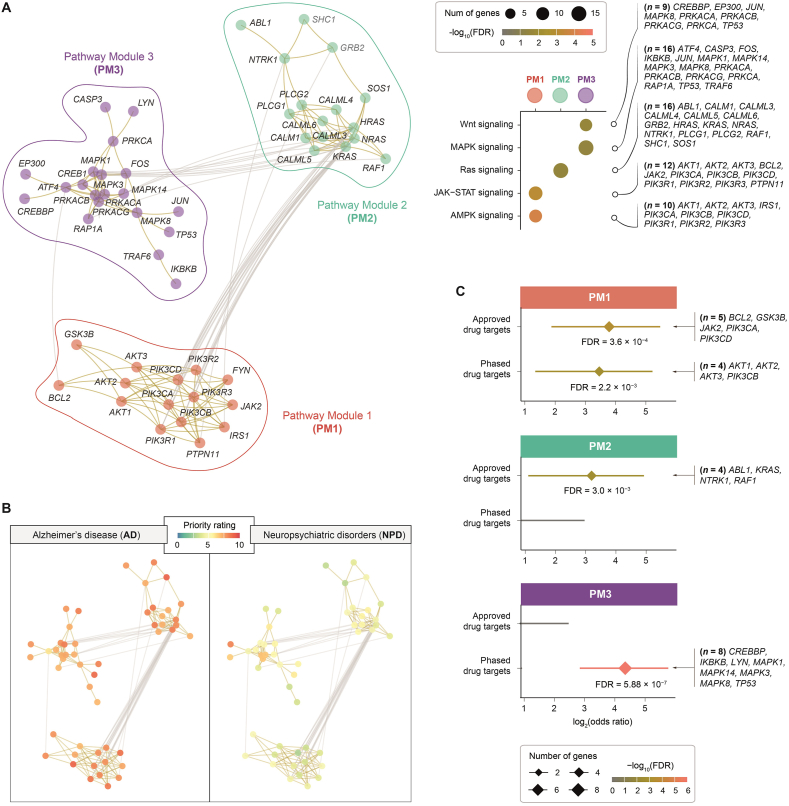
Crosstalk-based modular analysis for Alzheimer's disease (AD). Three pathway modules (PM1–PM3) were identified within the 51-gene crosstalk network *via* a spin-glass model and simulated annealing. Enrichment analysis was performed using a one-sided Fisher's exact test to calculate significance (false discovery rate [FDR]), odds ratio, and 95% confidence interval. (A) Modular visualisation of the 51-gene crosstalk network. Modules and member genes are colour-coded, with top KEGG pathway enrichments displayed in a dot plot (right panel). This visualisation identifies and characterises modules and their biological pathways, highlighting functional organisation and therapeutic relevance of the crosstalk network. (B) Disease-specific view of the crosstalk network. The same network layout as in (A) is presented, with nodes colour-coded by priority ratings in AD (left panel) *versus* neuropsychiatric disorders (NPD; right panel). This comparison reveals differentially prioritised genes and disease-specific mechanisms. (C) Forest plot comparing enrichments of approved drug targets and non-approved phased drug targets (sourced from ChEMBL) across modules. This plot provides a visual comparison of the enrichments across pathway modules, illustrating therapeutic potential.

We proceeded to explore the modular structure of the crosstalk network, identifying three distinct pathway modules (PM1–PM3), each intricately linked to specific molecular pathways (Fig. 6A). When colour-coded by priority rating (Fig. 6B), PM1 and PM2 were enriched for AD-specific genes with high prioritisation ratings, whereas PM3 showed relatively higher scores for AD, although some genes (such as *EP300* and *CREBBP*) were also highly prioritised in NPD (*i*.*e*., dual relevance to AD and NPD). Clinical evidence (approved drugs) strongly supported AD-specific pathway modules (PM1 and PM2), whereas PM3 showed preclinical potential *via* non-approved phased drug targets (Fig. 6C). Collectively, our crosstalk analysis positions Ras signalling as a linchpin of AD-specific pathway interactions, diverging from NPD-associated mechanisms. These insights underscore Ras signalling's potential as a therapeutic axis, complementing traditional amyloid/tau paradigms.

### 3.4 Literature support of AD-targeting molecular pathways centred on Ras signalling

PI4AD's identification of Ras signalling as a therapeutic hub in 10.13039/100020014AD is strongly supported by existing literature, which links key pathway components to 10.13039/100020014AD mechanisms. Below, we synthesise evidence for prioritised genes within this network (namely, *ABL1*, *AKT1-3*, *HRAS*, *KRAS*, *MAPK1-3*, *MAPK8*, *NTRK1*, *PIK3CA*, *PIK3CD*, *PRKCA*, *RAF1*, and *RAP1A*), highlighting their therapeutic relevance.

*ABL1 and AKT family. ABL1* strongly correlates with AD cerebrospinal fluid biomarkers and cognitive decline^69^, with ongoing phase-III trials (*e*.*g*., nilotinib) exploring its modulation in AD pathology^70^. The genes *AKT1-3*, particularly *AKT1* (targeted in breast cancer using capivasertib^71^), mediate crosstalk within Ras signalling, influencing pathways critical to AD progression.

*Ras GTPases (HRAS and KRAS).* While *HRAS* lacks extensive AD- or neuro-related literature, its central role in Ras signalling positions it as a candidate for mechanistic investigation. *KRAS*, validated in oncology (*e*.*g*., sotorasib for *KRAS* p.G12C-mutant lung cancer^72^), emerged as a compelling AD target due to its pathway centrality, despite the absence of AD-focused repurposing trials.

*MAPK family (MAPK1-3 and MAPK8). MAPK1-3* are targeted by oncology drugs such as dordaviprone and ulixertinib, which are actively being explored in cancer treatment^73^^,^^74^, while *MAPK8* is implicated in neuropathic pain^75^. Their roles in Ras signalling underscore cross-disease therapeutic potential.

*PI3K and downstream effectors (PIK3CA, PIK3CD,* and *RAF1). PIK3CA* and *PIK3CD*—linked to neuronal hyperactivity during glioma formation^76^ and primary immunodeficiencies^77^, respectively—may drive AD *via* Ras–PI3K–AKT axis dysregulation. *RAF1*, with well-characterised regulatory mechanisms^78^, provides a basis for targeted modulation in AD, even though there are currently no AD-specific drug repurposing trials.

*NTRK1 and PRKCA. NTRK1*, though unexplored in AD-focused drug repurposing trials, has immune-related roles in cancer^79^, aligning with Ras signalling's neuroinflammatory functions. *PRKCA*, despite limited AD literature, warrants mechanistic investigation in AD pathology due to its integration with Ras-centred signalling pathway.

*Ras–APP crosstalk.* Emerging evidence links Ras signalling to *APP* processing, both promoting the development of *APP* for AD and modulating the neuroprotection of *APP*^80^, ^81^, ^82^, ^83^, ^84^, ^85^, ^86^, ^87^. Recent findings associate the RAS/MAPK/ERK pathway with longitudinal AD biomarker changes^88^, further validating Ras signalling as a therapeutic nexus.

*Implications for therapeutic development.* PI4AD's integration of literature and multi-omics data positions Ras signalling as a priority axis for AD therapy. Repurposing oncology drugs (*e*.*g*., KRAS/PI3K inhibitors) or developing novel agents against *HRAS* or *MAPK8* could disrupt AD progression. These findings align with PI4AD's broader goal of bridging genetic insights to actionable pathway modulation, offering a roadmap for targeted AD therapeutics.

## 4 Discussion and conclusions

Our PI4AD framework represents a promising solution to bridge genetic insights and therapeutic discovery in AD. First, the recovery of clinical proof-of-concept targets such as *APP* and *ESR1* validates the prioritisation framework's efficacy. This not only reinforces methodological confidence but also underscores the pivotal roles of these genes in AD pathophysiology, which are actively pursued in drug development. Targeting these genes may yield therapies addressing specific AD mechanisms. Second, the construction of a self-organising prioritisation map delineates disease-specific targets in AD relative to neuropsychiatric disorders. By identifying distinct clusters, we elucidate AD's unique genetic architecture, guiding the development of precise therapeutic strategies. Finally, our exploration of pathway crosstalk highlights Ras signalling as a central hub, with genes such as *HRAS* and *MAPK1* emerging as high-priority targets. This systems-driven framework uncovers critical nodal points and drug-repurposing opportunities, deepening our understanding of AD pathogenesis.

While PI4AD builds on our previously established systems biology frameworks, its novelty lies in integrating multi-omics data with clinical translation. Diverging from traditional amyloid-*β*/tau-centric approaches, PI4AD prioritises Ras-centred pathway crosstalk—a shift supported by recent evidence linking Ras signalling to synaptic dysfunction and neuroinflammation. For instance, *HRAS* overexpression in the AD prefrontal cortex and *MAPK1*-mediated neuronal apoptosis validate PI4AD's target prioritisation. This holistic strategy addresses AD's multifactorial pathology, complementing singular pathway-focused therapies.

Benchmarking PI4AD against established tools (*e*.*g*., AlzGPS^15^ and Open Targets^42^) demonstrated competitive performance in recovering clinically relevant targets, alongside robustness confirmed *via* leave-one-out testing. While parameter tuning relied on clinical proof-of-concept targets to optimise biological relevance, this aligns with translational medicine guidelines, ensuring prioritisation reflects actionable therapeutic hypotheses rather than purely statistical associations.

The selection of NPDs as comparators, despite symptomatic overlaps, revealed divergent pathway activation: AD-specific Ras/MAPK signalling *versus* NPD-associated dopaminergic pathways. This aligns with genetic studies showing distinct dysregulation despite comorbidity^89^. Furthermore, focusing on clinical proof-of-concept targets (*e*.*g*., *APP* and *ESR1*) reflects their validation in human trials^90^, addressing historical drug failures attributed to non-genetic or non-human targets^91^.

PI4AD's identification of Ras signalling as a therapeutic hub marks a departure from conventional paradigms. Ras pathways mediate critical AD processes—neuroinflammation and synaptic plasticity—through crosstalk with amyloid-*β*/Tau-independent mechanisms. For example, *HRAS* modulates microglial activation *via* NF-*κ*B, while *MAPK1* regulates neuronal apoptosis in AD models. Prioritising these targets addresses the limitations of singular amyloid/tau focus, offering a multi-faceted strategy to disrupt AD progression.

Recent advances in ANNs, such as PALH^30^ and DGCL^31^, excel in neuroimaging biomarker discovery. PI4AD complements these by prioritising therapeutic targets through multi-omics integration (GWAS, QTL, and PCHi-C) and pathway crosstalk analysis, bridging biomarker discovery with therapeutic development. This distinction underscores the versatility of ANNs in AD research, connecting neuroimaging-driven biomarker discovery with systems biology-led therapeutic development.

*Limitations and future directions*. A key limitation of this study is the reliance on European-centric GWAS data, which may introduce population-specific biases and limit generalisability of prioritised targets. While this reflects current AD genetic data availability, future iterations will incorporate diverse cohorts (*e*.*g*., Asian and African ancestries) and ancestry-specific heritability estimates, aligning with global equity initiatives to diversify genomic resources in neurodegenerative research^92^.

*Translational roadmap and broader implications*. To translate PI4AD's computational medicine insights into clinical impact, we propose: (i) *in vitro* validation of high-priority Ras pathway targets (*e*.*g*., *HRAS* and *MAPK1*) using AD patient-derived induced pluripotent stem cell models to assess their role in amyloid-*β* and tau pathology^93^; and (ii) clinical partnerships to evaluate Ras-modulating drugs (*e*.*g*., KRAS inhibitors^72^) or implement mRNA therapeutics with lipid nanoparticles (as demonstrated for amyloid-*β*^5^^,^^94^), accelerating pathway-targeted therapies. Beyond AD, PI4AD's framework holds broader implications. First, its application in AD could extend to Parkinson's disease or Huntington's disease, leveraging multi-omics and network evidence for therapeutic discovery. Second, Ras signalling's role in neuroinflammation^95^ positions it as a multi-target therapeutic hub. Lastly but not the least, the use of ANNs and advanced computational methods encourages adoption in other biomedical fields. In conclusion, PI4AD advances therapeutic discovery by unifying systems biology with translational application. By prioritising Ras-centred crosstalk, benchmarking against existing tools, and proposing actionable validation, it addresses historical challenges in neurodegenerative drug development, paving the way for transformative therapies.

## Author contributions

Yisheng Yang: Data curation, Investigation, Methodology, Writing – original draft. Yizhu Diao: Investigation, Writing – original draft. Lulu Jiang: Conceptualisation, Writing – original draft. Fanlu Li: Writing – review & editing. Liye Chen: Writing – review & editing. Ming Ni: Funding acquisition, Resources, Writing – review & editing. Zheng Wang: Funding acquisition, Resources, Writing – review & editing. Hai Fang: Conceptualisation, Data curation, Funding acquisition, Investigation, Methodology, Project administration, Resources, Software, Supervision, Visualisation, Writing – original draft.

## Conflicts of interest

The authors declare no competing interests.

## Appendix A Supporting information

The following is the Supporting Information to this article.
