# Supplementary material for: A computational medicine framework integrating multi-omics, systems biology, and artificial neural networks for Alzheimer's disease therapeutic discovery
Source: Acta Pharm Sin B. 2025 Jul 16;15(9):4411–26. doi: 10.1016/j.apsb.2025.07.018 (PMC12491700; doi:10.1016/j.apsb.2025.07.018)
Supplement: Multimedia component 1 [file mmc1.pdf]

Supporting Information for

Original article

A computational medicine framework integrating multi-omics, systems biology, and artificial neural networks for Alzheimer's disease therapeutic discovery

Yisheng Yang[a,†], Yizhu Diao[a,†], Lulu Jiang[b,†], Fanlu Li[c], Liye Chen[d], Ming Ni[e,*], Zheng Wang[f,g,h,*], Hai Fang[a,*]

[a]*Shanghai Institute of Hematology, State Key Laboratory of Medical Genomics, National Research Center for Translational Medicine at Shanghai, Ruijin Hospital, Shanghai Jiao Tong University School of Medicine, Shanghai 200025, China*

[b]*Translational Health Sciences, University of Bristol, Bristol BS1 3NY, UK*

[c]*Department of General Surgery, Pancreatic Disease Center, Ruijin Hospital, Shanghai Jiao Tong University School of Medicine, Shanghai 200025, China*

[d]*Nuffield Department of Orthopaedics, Rheumatology and Musculoskeletal Sciences, University of Oxford, Oxford OX3 7LD, UK*

[e]*Department of Orthopaedics, Shanghai Key Laboratory for Prevention and Treatment of Bone and Joint Diseases, Shanghai Institute of Traumatology and Orthopaedics, Ruijin Hospital, Shanghai Jiao Tong University School of Medicine, Shanghai 200025, China*

[f]*Jinfeng Laboratory, Chongqing 401329, China*

[g]*Medical Center of Hematology, Xinqiao Hospital of Army Medical University, State Key Laboratory of Trauma and Chemical Poisoning, Chongqing Key Laboratory of Hematology and Microenvironment, Chongqing 400037, China*

[h]*Bio-Med Informatics Research Center & Clinical Research Center, the Second Affiliated Hospital, Army Medical University, Chongqing 400037, China*

Received 6 December 2024; received in revised form 27 May 2025; accepted 27 May 2025

[*]Corresponding authors. gendianqing@163.com (Ming Ni), biowz@mail.ustc.edu.cn (Zheng Wang), fh12355@rjh.com.cn (Hai Fang).

[†]These authors made equal contributions to this work.

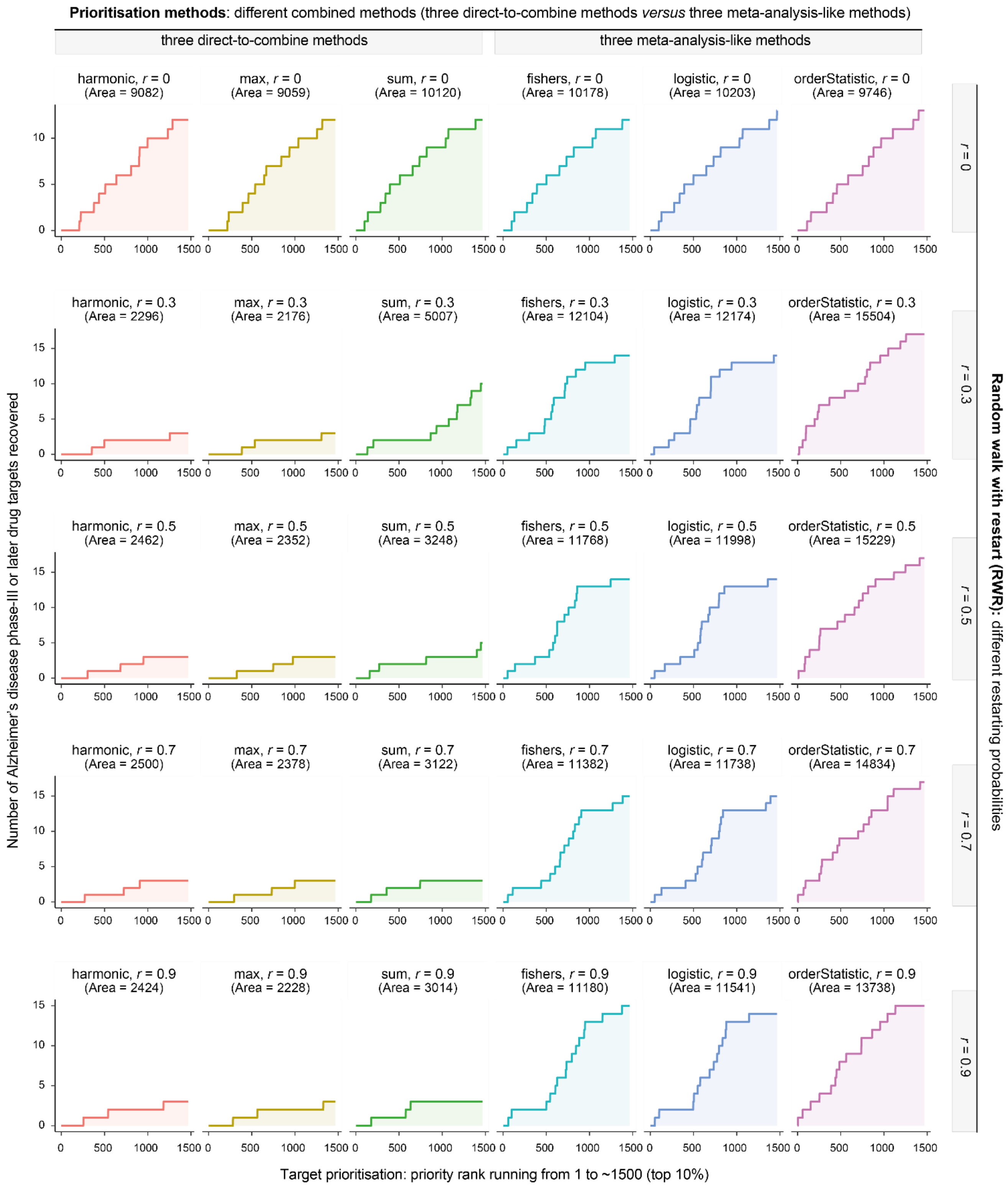

Figure S1 Optimisation analysis for recovering clinical proof-of-concept targets for Alzheimer's disease (AD). The figure evaluates combined meta-analysis-like methods and random walk with restart (RWR) restarting probabilities for recovering pre-existing phase-III or later AD drug targets. Columns denote combined methods, and rows illustrate RWR restarting probabilities. Optimal performance

(highlighted) corresponds to the order statistic method with $\gamma = 0.3$, critical for developing accurate target prioritisation strategies.

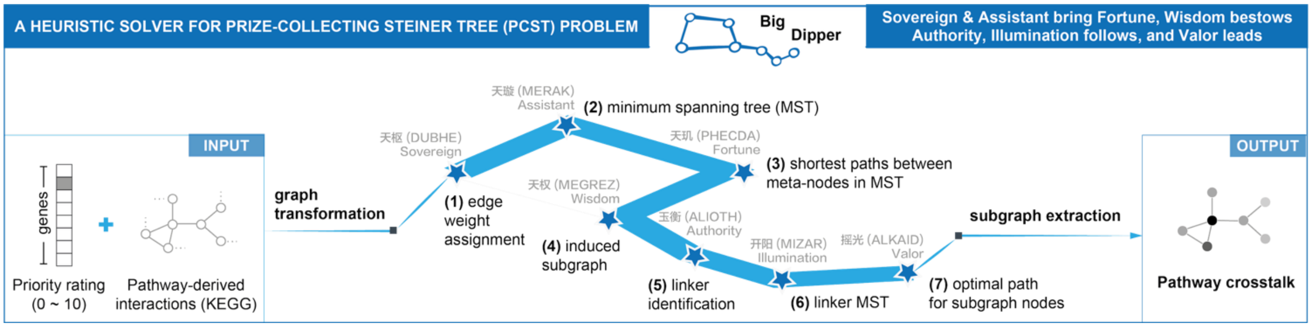

Figure S2 Prize-collecting Steiner tree (PCST) workflow. Inspired by Bip Dipper, this PCST workflow outlines pathway crosstalk identification process, from initial graph transformation to final subgraph extraction spanning seven steps between-in. (0) Graph transformation: combine connected positive nodes into isolated meta-nodes, link these meta-nodes *via* negative single-nodes, and assign positive scores to meta-nodes and negative scores to single-nodes. (1) Edge weight assignment: add weights to edges in the transformed graph, define two types of edges including single-single edges (connecting two single-nodes with weights based on the absolute sum of their scores normalised by degrees) and single-meta edges (connecting a single-node and a meta-node with weights based on the absolute score in the single-node normalised by its degree), and ensure all weights are non-negative. (2) Minimum spanning tree (MST): use Prim's greedy algorithm to find MST in the weighted transformed graph, that is, a minimised-edge-weight subgraph that connects all nodes. (3) Shortest paths between meta-nodes: find all shortest paths between any pair of meta-nodes in MST. (4) Induced subgraph: create a subgraph with nodes from the shortest paths and edges between them. (5) Identification of linkers: identify single-nodes directly connected to meta-nodes in a manner that each identified single-node must have absolute scores no greater than the sum of scores in its neighboring meta-nodes, and these quantified single-nodes are called 'linkers'. (6) Linker MST: find MST in the linker graph containing only linkers and their connecting edges. (7) Optimal path and subgraph nodes: from the linker MST and attached meta-nodes, find the optimal path maximising the sum of scores for nodes and attached meta-nodes among all possible paths, and nodes along this optimal path and their attached meta-nodes are termed 'subgraph nodes'. Finally, extract a subgraph from the input graph containing only subgraph nodes and edges connecting them; this subgraph (or 'pathway crosstalk') represents the maximum-scoring subgraph with as many positive nodes as possible, minimising the inclusion of negative nodes.

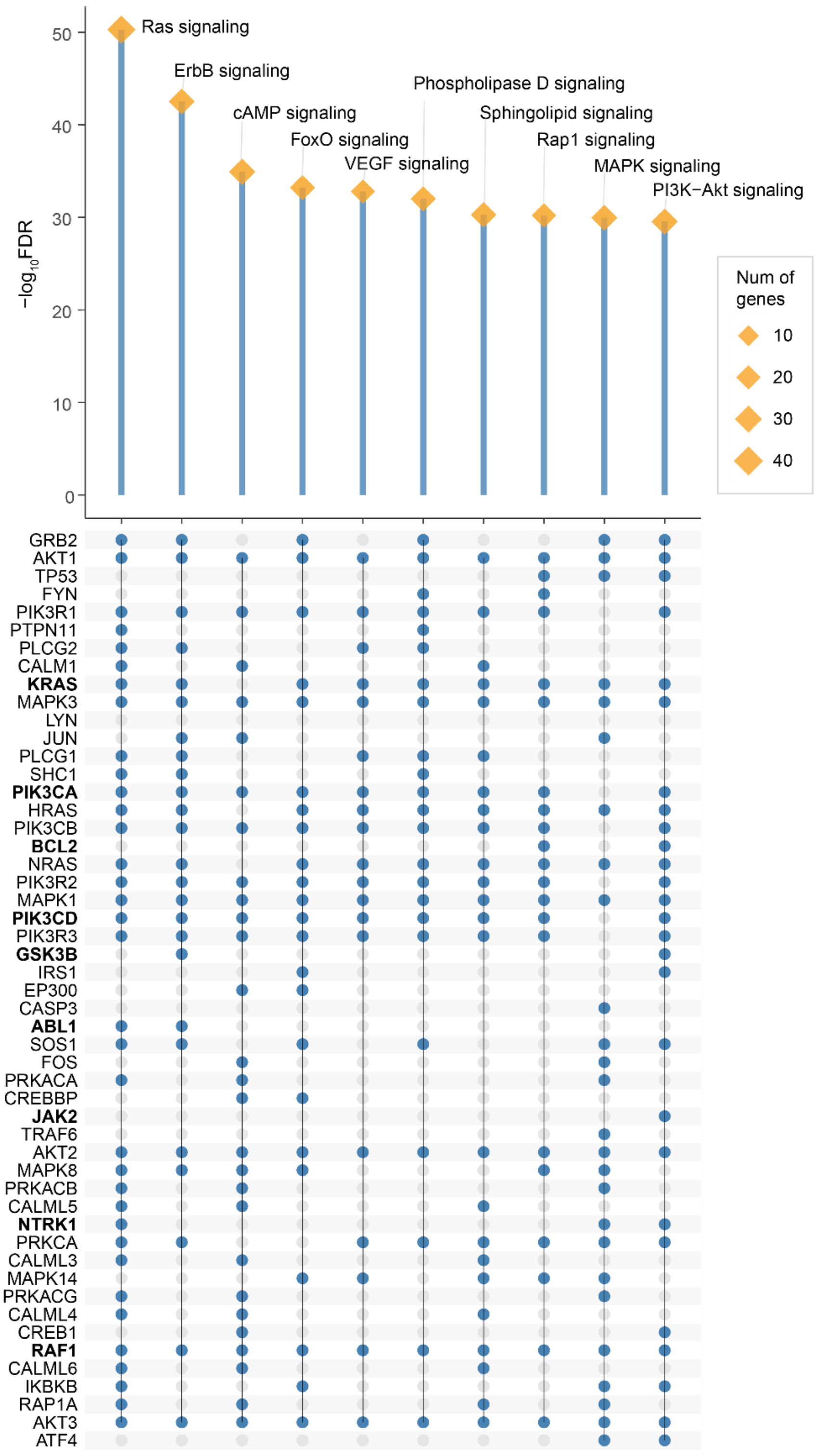

Figure S3 KEGG pathways enriched in crosstalk genes. Enrichment significance (false discovery rate [FDR]) was calculated using a one-sided Fisher's exact test. Kite size corresponds to the number of member genes, as indicated by blue dots beneath. This visualisation highlights overrepresented pathways, guiding investigations into molecular mechanisms and therapeutic targets.

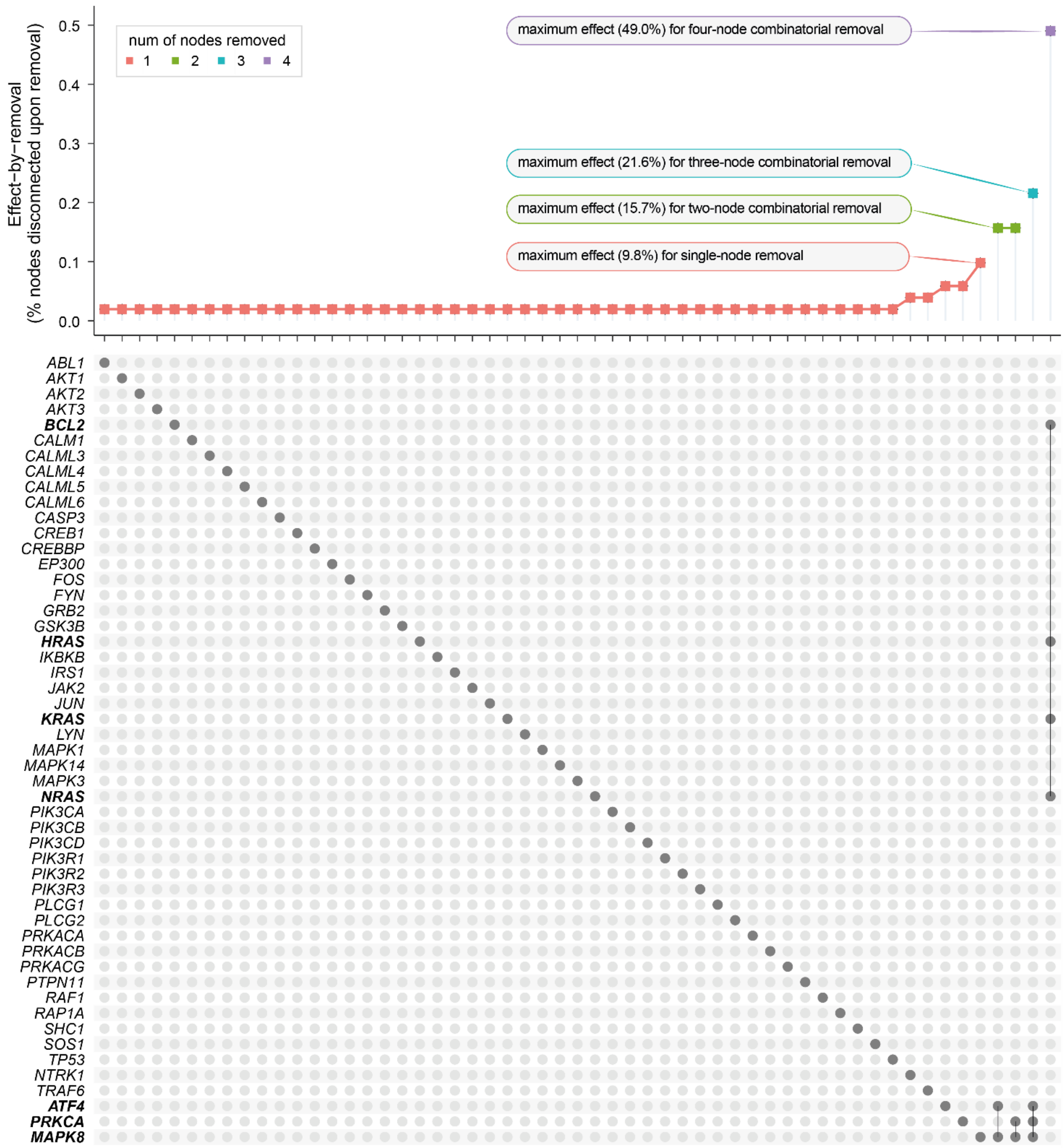

Figure S4 Crosstalk-based effect-by-removal analysis. The fraction of disconnected nodes (*y*-axis) is plotted against individual or combinatorial node removal (*x*-axis). Optimal combinatorial removals (two-, three-, and four-node) with maximal effects are illustrated. This identifies critical nodes, informing therapeutic strategies to disrupt crosstalk and disease progression.
